# Beyond meaning: hope and secondary trauma in Israeli therapists after the October 7th massacre

**DOI:** 10.3389/fpsyg.2025.1594885

**Published:** 2025-06-04

**Authors:** Sivan George-Levi, Lir Faverman, Yael Galin-Lonchich, Anat Ben-Gal Dahan, Rivi Frei-Landau

**Affiliations:** ^1^Department of Psychology, Achva Academic College, Arugot, Israel; ^2^Israeli Center for Addiction and Mental Health (ICAMH), Department of Psychology, The Hebrew University of Jerusalem, Jerusalem, Israel; ^3^Sderot Resilience Center, Sderot, Israel

**Keywords:** hope, meaning, secondary trauma, therapists, war

## Abstract

The massacre on October 7th, 2023 in southern Israel had a profound impact on mental health therapists in the region. Such collective trauma can lead to heightened stress and secondary trauma among therapists. Identifying resilience and risk factors is, therefore, essential for mitigating these effects. This preliminary study involved 60 therapists (75% women; *M* = 48.3 years, *SD* = 10.7) from the Sderot Resilience Center, located near the Gaza border, who completed questionnaires about demographic, professional, and war-related factors, as well as secondary trauma symptoms, finding meaning in work, sense of hope, and stress levels during the war, 1 year after the attack. Loss of a loved one during the war was associated with higher stress levels. Secondary trauma symptoms were linked with increased stress, and higher levels of hope were associated with reduced stress. Moderation models indicated that finding meaning in work was associated with lower stress only when secondary trauma symptoms were low or moderate, but not when they were high. Higher hope levels were linked to reduced stress regardless of secondary trauma severity. Although preliminary and cross-sectional, these findings suggest that whereas finding meaning in work may motivate therapists, it may not fully protect them against stress during extreme trauma. Hope, however, appears to offer a stronger protective buffer. Interventions to mitigate risk factors, reduce secondary trauma, and foster hopeful thinking are essential.

## Introduction

The Hamas massacre in Israel on October 7th, 2023 significantly amplified the challenges faced by trauma therapists, particularly those working near the Gaza border (Taubman Ben Ari et al., 2024). Residents in this area endured direct threats to their lives, relentless rocket attacks, the destruction of their homes, and the heartbreaking loss or abduction of loved ones. In the aftermath of the massacre, many residents were evacuated from their homes and were compelled to grapple with profound trauma, uncertainty, and fear (Levi-Belz et al., 2024).

In the year following the massacre, trauma therapists working near the Gaza border confronted the dual burden of supporting clients deeply affected by the massacre and the war, while simultaneously enduring the same collective trauma themselves (Saar-Ashkenazy et al., 2024; Stahnke and Firestone, 2024). This phenomenon, known as “shared traumatic reality,” highlights the unique difficulty of being both personally impacted by a catastrophic event and professionally responsible for helping others cope with it (Baum, 2010).

A study conducted a month after the massacre found alarmingly high rates of post-traumatic stress disorder (PTSD) symptoms, depression, and anxiety among Israeli residents, reflecting the immense psychological impact of the October 7th atrocities on the population and the corresponding strain on mental health providers (Levi-Belz et al., 2024). Several studies (Ben-Kimhy et al., 2025; Erel-Brodsky et al., 2024; Taubman Ben Ari et al., 2024) revealed that therapists, like their clients, experienced intense feelings of helplessness, fear, sorrow, and a sense of a collapsing world. Yet many therapists sought to sustain their sense of meaning through their work as a way to cope. Mental health workers who experienced trauma prior to October 7th reported increased stress and secondary trauma shortly after the massacre, whereas personal resilience was associated with reduced stress among this cohort (Dahan et al., 2024).

According to conservation of resources (COR) theory (Hobfoll and Hou, 2025), under traumatic conditions, individuals strive to preserve resources essential for self-protection as a way to mitigate stress. Extreme events, such as the October 7th events in Israel, underscore the urgent need to identify risk and resilience factors that can effectively mitigate stress levels for therapists working in contexts of collective trauma, particularly in high-intensity war zones. Previous studies have shown that “finding meaning in work” and “sense of hope” act as resilience resources, helping to reduce stress among trauma therapists (Passmore et al., 2019). Although numerous resilience factors have been identified, recent research that was conducted after the attacks underscored the notion that these two factors are particularly crucial in fostering the well-being of trauma therapists (Lev et al., 2023; Taubman Ben Ari et al., 2024).

Guided by COR theory, in this exploratory study we explored how different resources such as demographic factors, professional factors, war-related factors, and personal resilience factors (i.e., finding meaning in work and sense of hope) were related to the stress levels of therapists working in a war zone (i.e., near the Gaza border in Israel), 1 year after the massacre. We also examined how personal resilience factors interacted with the risk factor of secondary trauma symptoms to mitigate therapists’ stress levels.

### Stress among trauma therapists

Trauma therapists are particularly vulnerable to stress and tension due to the emotionally demanding nature of their work (Molnar et al., 2017). Constant exposure to clients’ traumatic experiences can lead to greater stress, as therapists are highly engaged in the emotional distress of those they treat (Figley, 2002; Leung et al., 2023). The unpredictability of trauma work, ethical dilemmas, and the pressure to provide effective interventions contribute to this heightened stress, which can become chronic (Bride, 2004).

Recent studies have identified several key factors associated with trauma therapists’ stress. Demographic factors such as younger age, less clinical experience, and being female have been associated with their higher levels of stress (Cieslak et al., 2014). Therapists’ negative personal perspectives on treatment also influence stress outcomes (Miller Itay and Turliuc, 2023). Additionally, lack of resilience and inadequate self-care practices increase vulnerability to stress and burnout among therapists, particularly during crises (Cook and Fye, 2023; Litam et al., 2021). Active engagement in providing assistance has, for its part, been identified as a moderating factor in the relation between traumatic stress and resilience among first responders (Saar-Ashkenazy et al., 2024). Furthermore, personal and job-related factors (gender, resilience, social support, frequency of moral dilemma, and exposure to client violence), rather than years of experience, have also been found to influence stress among trauma therapists (Lev et al., 2022).

### Secondary trauma

Studies suggest that a major factor in trauma therapists’ stress is continuous exposure to clients’ traumatic experiences, which can lead to secondary trauma symptoms and emotional exhaustion (Leung et al., 2023). Secondary trauma symptoms include symptoms such as intrusive thoughts, flashbacks, and nightmares (Gentry, 2002), which may lead to distress indicators such as sleep disturbances, emotional withdrawal, irritability, and difficulty concentrating (Finzi-Dottan and Berckovitch Kormosh, 2016; Hamama-Raz and Minerbi, 2019; Quinn et al., 2019). Such symptoms may also lead to neglect or minimization of client needs (Finzi-Dottan and Berckovitch Kormosh, 2016).

Some therapists may even develop secondary traumatic stress (STS), a condition that closely resembles PTSD, due to repeated exposure to clients’ traumatic experiences (Leung et al., 2023). When left unaddressed, STS can escalate, increasing the risk of developing a PTSD diagnosis and impairing therapists’ emotional well-being, professional functioning, and overall quality of life (Hensel et al., 2015).

### Meaning in work

Meaning in work is defined as individuals’ subjective perception of significance and value in their work (Allan et al., 2015). Martela and Pessi (2018) defined meaningful work as consisting of the intrinsic value of the work itself, self-realization, and the contribution one feels one is making to a larger purpose. Meaningful work is strongly associated with work engagement, commitment, and job satisfaction, as well as with overall well-being and reduced stress (Allan et al., 2019). The more therapists find meaning in their work, the lower their levels of burnout are likely to be, even when their work involves heavy workloads and is performed under challenging conditions (Pardes et al., 2014).

Taubman Ben Ari et al. (2024) found that therapists responding to the aftermath of October 7th in Israel balanced the stress of working with clients by deriving meaning from their work. They reported positive outcomes such as pride, accomplishment, personal and spiritual growth, and renewed purpose. Levkovich and Labes (2024) indicated that counselors working in Israeli communities along the Gaza border experienced a deep sense of fulfillment and positive psychological growth, which they attributed to the meaningful nature of their work with evacuees (i.e., from the communities that, after the October 7th attack, were at further risk of war or had been destroyed). Accordingly, one of the recommendations of the National Council for Post-Trauma following the events of October 7th was to cultivate a sense of meaning in work as a protective factor for first responder therapists (Lev et al., 2023), which provided the rationale for focusing on meaning in work in our study.

### Hope

According to Snyder (2002), hope represents the ability to identify pathways toward desired goals and to motivate oneself to pursue those paths. Hope is considered to be a significant predictor of individual psychological adjustment in challenging life situations (Deniz et al., 2023; Gallagher et al., 2020). Studies have indicated that hopeful individuals are also more skilled at identifying effective strategies to achieve their goals and navigate challenges, making it easier for them to cope with and manage stress (Shorey et al., 2007).

Lev et al. (2023) emphasized the crucial role of hope in enhancing resilience among therapists. Consistently, hope has been identified as a key protective factor for professionals working in high-stress environments (Passmore et al., 2019; Pharris et al., 2022; Usta and Bekircan, 2024). Sustaining hope not only supports therapists’ well-being and reinforces their sense of purpose and commitment to their work, but also benefits clients by fostering their post-traumatic growth (Levi, 2013). Given the demonstrated importance of hope in therapeutic resilience, we chose to focus on this variable in the current study to explore its contribution to therapists’ reduced stress levels.

### The current study

Trauma therapists’ demanding role during a collective trauma has been shown to heighten their vulnerability to stress. In this exploratory study we focused on therapists working at the Sderot Resilience Center in Israel 1 year after the October 7th massacre. Sderot is the largest city near the Gaza border, which has long faced ongoing exposure to traumatic events. On October 7th Sderot suffered a brutal attack, forcing all residents to evacuate from their homes for months. The Sderot Resilience Center, which has provided emotional support to the Sderot community for over 15 years, intensified its efforts after the attacks, with both new and experienced therapists joining forces to address the increased demand for care.

In accordance with COR theory, we explored the connection between the therapists’ demographic and professional aspects, war-related factors, and resilience factors, and the degree to which the therapists felt stress. Specifically, we hypothesized that finding meaning in work as well as sense of hope would serve as resilience factors, and that therapists who experienced high levels of meaning and hope would report lower levels of stress. However, given that these therapists worked in a high-intensity war and traumatic zone, we proposed that the protective effect of these resilience factors might depend on the severity of their secondary trauma symptoms. In other words, we assumed that varying levels of secondary trauma symptoms might moderate the extent to which meaning and hope would mitigate therapists’ stress.

## Materials and methods

### Participants

Eligible participants consisted of 258 trauma therapists affiliated with the Sderot Resilience Center, located near the Gaza border, 1 year following the October 7th attack. All eligible individuals were invited to participate via a targeted outreach in professional online groups. Of those approached, 72 therapists provided informed consent to participate, and 60 completed all study questionnaires in full. Of the therapists, 75% were women. Therapists’ average age was 48.2 years (*SD* = 10.7) and ranged from 30 to 74 years. The majority (77.8%) were married or in a relationship, and had an average of 2.93 children (*SD* = 1.5). Regarding religious affiliation, 58.3% identified as secular, 8.3% as traditional, 20% as religious, and 3.3% as other. Participants had an average of 17.7 years of education (*SD* = 3.5).

Therapists’ average professional experience was 15 years in duration (*SD* = 8.8). Concerning their professional tenure at the Sderot Resilience Center, 50% had been working there for 1 year, 24.1% for 1–4 years, 14.8% for 5–10 years, and 11.1% for more than 10 years. On average, study participants treated 12.07 clients per week (*SD* = 6.5). Geographically, 18.5% resided in the Gaza Envelope (i.e., the populated areas in the Southern District of Israel that are within seven kilometers of the Gaza Strip border and are therefore within range of mortar shells and Qassam rockets launched from the Gaza Strip); 33.3% lived within a 40-kilometer range of the Gaza border; and 48.1% lived more than 40 kilometers away. Additionally, 20.4% of the participants were evacuated from their homes during the Swords of Iron War – namely, the Israel-Hamas war that began following the October 7th Hamas attack on Israel—and 24.1% reported experiencing the loss of a loved one during the war.

### Instruments

#### The work and meaning inventory (WAMI)

Developed by Steger et al. (2012), the WAMI consists of 10 items divided into three subscales: positive meaning, meaning through work, and motivation for the common good. For this study, the “motivation-for-the-common-good” subscale was used to assess participants’ sense of finding meaning in their work. Respondents rated each item on a 7-point Likert scale. In this study, we adjusted the questionnaire to address participants’ work at the Resilience Center. An example item was: “I know that my work at the Resilience Center makes a positive change in the world.” The Hebrew version of the scale has been used in previous studies (e.g., Yakobi et al., 2023). Cronbach’s alpha coefficient was 0.65.

#### Compassion fatigue scale-revised

This 13-item self-report questionnaire, developed by Adams et al. (2006), includes two subscales: (1) a five-item secondary trauma subscale, and (2) an eight-item work burnout subscale. Respondents rate each item on a 10-point Likert scale, from 1 (*never or rarely*) to 10 (*very often*). In the current study, we focused on the secondary trauma subscale. An example item from the secondary trauma subscale was: “I experienced disturbing dreams similar to those of my clients.” The ratings for all items from the secondary trauma subscale are summed, and the average of these sums represents the final score. Cronbach’s alpha coefficient was 0.75.

#### The Hope Scale

The Hebrew version of the Hope Scale (Lackaye and Margalit, 2006), originally developed by Snyder (2002), is a self-report measure for adults that assesses hope levels. It consists of six statements, answered on a 6-point scale, with the hope score calculated as the average of the responses. The items are divided into two subscales: goal-oriented thinking (e.g., “I feel that I am achieving the goals I have set for myself”) and process-oriented thinking (e.g., “If I encounter a problem, I can think of many ways to resolve it”). Cronbach’s alpha coefficient was 0.86.

#### Stress

Participants’ stress levels were assessed using one question: “In the past few weeks how much stress/tension did you experience?” Responses ranged from 1 (*rarely*) to 7 (*very often*). The use of one question to assess stress, in order to reduce the burden on participants, has been validated in former studies (Elo et al., 2003; Littman et al., 2006).

#### Sociodemographic data

Participants were asked to complete a short demographic questionnaire regarding gender, age, years of education, marital status, religiosity, area of residence, length of time working at the Resilience Center, how qualified they felt to work with trauma (1 = *not at all* to 7 = *very much*), number of clients, and the impact of the Swords of Iron War. This last aspect included questions regarding participants’ evacuation from their homes and any loss of a loved one they experienced during the war (i.e., personal loss).

### Procedure

After receiving approval from the authors’ college’s ethics committee and the Sderot Resilience Center, the questionnaires were presented as an electronic link to therapists working at the Sderot Resilience Center by the research team. Missing data accounted for ~3.3% of the sample and were handled using available-case analysis, whereby cases with missing values on variables relevant to a given analysis were excluded. The preliminary analysis consisted of t-tests, analyses of variance (ANOVAs), and Pearson correlations using SPSS 29 in order to examine associations among the demographic and research measures. Based on theorized methods in which secondary trauma symptoms may moderate the relation between finding meaning in work and stress, and between sense of hope and stress, a moderation model was examined, using Preacher and Hayes’ (2008) bootstrapping method with 5,000 resamples with replacement. Bootstrapping was used as it provides a more reliable estimate of indirect effects and does not assume normality (Preacher and Hayes, 2008).

### Sample size calculation

A power analysis was conducted using GPower 3.1 to determine the required sample size for a multiple regression analysis with four predictors, including the interaction term for moderation. The analysis was based on an alpha level of 0.05, a power of 0.80, and effect size estimates following Cohen’s (1988) guidelines: small (f^2^ = 0.02), medium (f^2^ = 0.15), and large (f^2^ = 0.35). The required sample size for a medium effect was 77, and for a large effect it was 36.

## Results

### Associations between the study variables

Therapists’ stress levels did not differ in terms of gender [*t*_(58)_ = 1.52, *p* = 0.07], evacuation status [*t*_(58)_ = −1.39, *p* = 0.73], geographic residence [*F*_(2,57)_ = 1.57, *p* = 0.21, *η*2 = 0.05], or religion [*F*_(3,56)_ = 1.25, *p* = 0.30, *η*2 = 0.07]. Therapists who experienced personal loss during the war reported higher stress levels than those who had not experienced such a loss [*t*_(58)_ = −2.55, *p* = 0.00]. The effect size, as measured by Cohen’s d, was large, 𝑑= − 0.81.

Pearson correlations did not indicate a significant correlation between stress and age (*r* = −0.07, *p* = 0.58), experience in trauma therapy (*r* = −0.17, *p* = 0.22), number of clients (*r* = −0.13, *p* = 0.31), or length of time working at the Resilience Center (*r* = 0.22, *p* = 0.10).

Means and standard deviations (*SD*s) and Pearson correlations among the research measures are presented in Table 1. As can be seen in Table 1, stress was significantly and positively correlated with secondary trauma symptoms and negatively correlated with sense of hope. No significant correlation was found between the variables and finding meaning in work. A moderation analysis was performed to further examine the interactions between the predictors.

**Table 1:** Means, *SD*s, and Pearson correlations among the variables.

Variables	1	2	3	4
1. Stress	--			
2. Secondary trauma	0.36**	--		
3. Hope	−0.31*	−0.19	--	
4. Meaning	−0.02	−0.11	0.07	--
M	4.21	12.08	4.31	5.55
*SD*	1.51	4.85	0.62	0.88

*p < 0.05; **p < 0.01. The variables in the table are numbered as follows: 1 = Stress, 2 = Secondary trauma, 3 = Hope, 4 = Meaning.

### Moderation analyses

To assess the robustness of these findings and determine whether the relation between finding meaning in work and stress varied across different levels of secondary trauma symptoms (at the 16th, 50th, and 84th percentiles), a moderation model (Command Model 1) was tested using SPSS PROCESS macro (Hayes, 2018). Interaction terms were calculated using mean-centering, and personal loss during the war was included as a covariate in the analysis.

**Figure 1:**
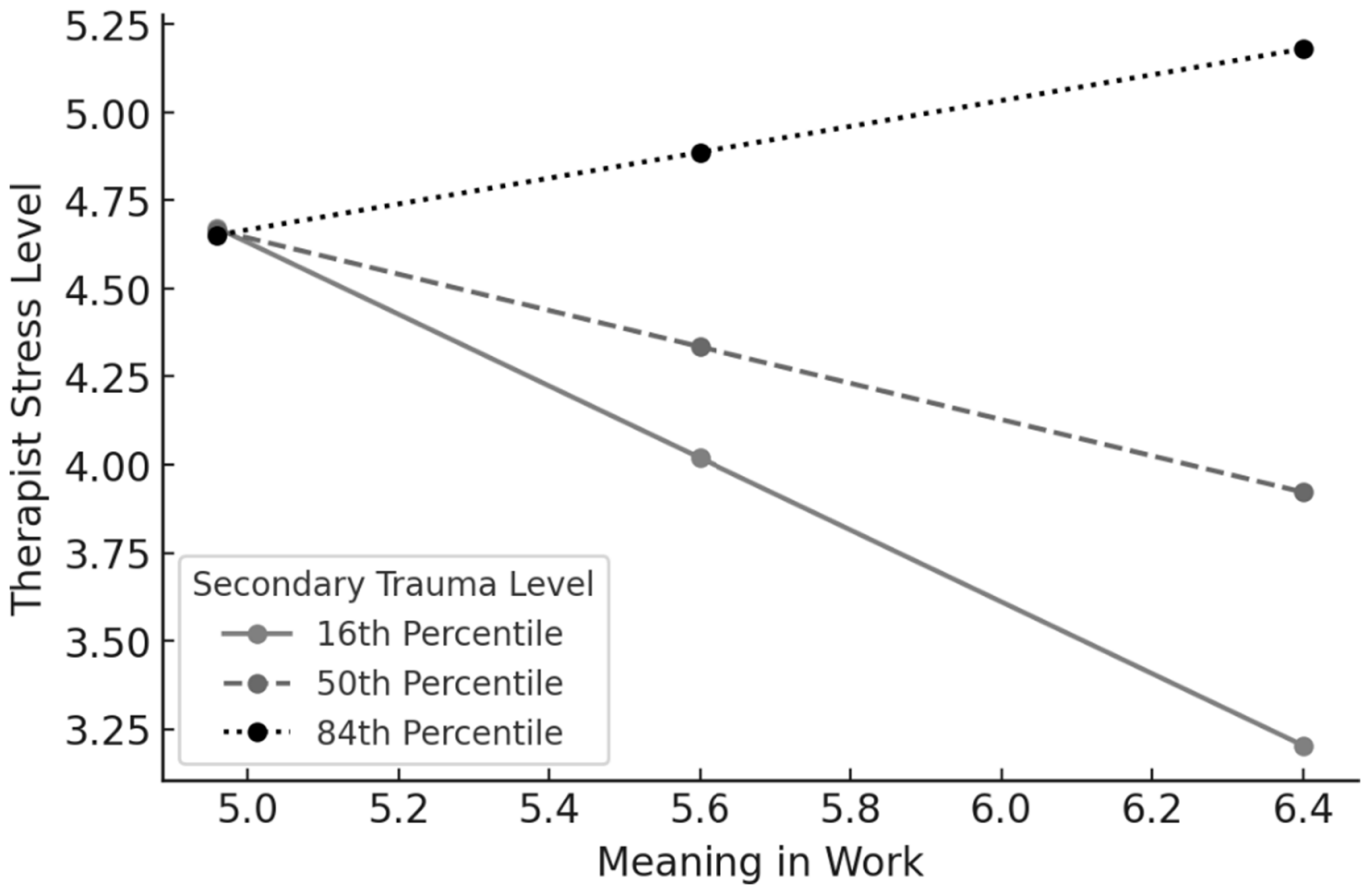
Secondary trauma symptoms moderate the relation between finding meaning in work and stress.

The model was significant, *F*_(4,55)_ = 4.77, *p* = 0.00, explaining 28.03% of the variance in stress (Cohen’s f^2^ = 0.39), indicating a large effect. The model indicated that finding meaning in work was significantly related to stress, *b* = −1.90, *SE* = 0.72, *t* = −2.61, *p* = 0.01, 95% CI = [−3.3657, −0.4410]. Secondary trauma symptoms were significantly related to stress, *b* = −3.13, *SE* = 1.33, *t* = −2.35, *p* = 0.02, 95% CI = [−5.8102, −0.4634]. Furthermore, secondary trauma symptoms significantly moderated the relation between finding meaning and stress, *b* = 0.63, *SE* = 0.23, *t* = 2.67, *p* = 0.01, 95% CI = [0.1560, 1.1052]. Analysis of the moderation effect indicated that the relation between finding meaning and stress was significant for low levels of secondary trauma symptoms (16th percentile), *b* = −1.02, *SE* = 0.42, *t* = −2.41, *p* = 0.01, 95% CI = [−1.8686, −0.1724], but not for medium (50th percentile), *b*0- 0=.51, *SE* = 0.27, t = −1.87, *p* = 0.06, 95% CI = [−1.0681, 0.0361], or high (84th percentile) levels of secondary trauma, *b* = 0.36, *SE* = 0.26, *t* = 1.41, *p* = 0.16, 95% CI = [−0.1558, 0.8895]. In other words, finding meaning in work was negatively related to stress only when secondary trauma symptoms were low. The implication of this moderation is that stress was lower for therapists who found a lot of meaning in their work only when they experienced low levels of secondary trauma symptoms. When they experienced medium or high levels of secondary trauma symptoms, the protective effect of finding meaning in their work on stress was non-significant, as depicted in Figure 1.

A second moderation model was conducted in order to investigate whether secondary trauma symptoms moderated the relation between hope and stress, controlling for personal loss. The model was significant *F*_(4,55)_ = 7.07, *p* = 0.00, explaining 36.60% of the variance in stress (Cohen’s f^2=^ 0.57), indicating a large effect. The model indicated that hope was significantly related to stress, *b* = −1.59, *SE* = 0.72, *t* = −2.21, *p* = 0.03, 95% CI = [−3.0500, −0.1454]. Secondary trauma symptoms were not significantly related to stress, *b* = −0.66, *SE* = 1.37, *t* = −0.48, *p* = 0.63, 95% CI = [−3.4211, 2.1018]. Furthermore, secondary trauma symptoms did not significantly moderate the relation between hope and stress, *b* = 0.27, *SE* = 0.31, *t* = 0.86, *p* = 0.39, 95% CI = [−0.3600, 0.9021]. In other words, hope was negatively related to stress regardless of secondary trauma symptoms. The implication of this finding is that stress was lower for therapists with high levels of hope regardless of their secondary trauma levels.

## Discussion

The aim of this study was to investigate factors related to stress levels among trauma therapists working at the Sderot Resilience Center near the Gaza border 1 year after the Hamas massacre in Israel on October 7th, 2023, under the unique conditions of a collective trauma. No significant relations were found between therapists’ stress levels and their demographic or professional characteristics; the importance of war-related factors and psychological aspects was thus emphasized.

Overall, therapists reported relatively high levels of finding meaning in their work. These results align with prior research suggesting that mental health professionals often derive significant meaning from their work (Taubman Ben Ari et al., 2024). However, the results also indicated that only when secondary trauma symptoms were low was finding meaning in work linked to reduced stress. When secondary trauma symptoms were high, the beneficial effect of finding meaning in work diminished.

These results accord with COR theory (Hobfoll and Hou, 2025), which posits that stress arises when individuals perceive a threat to their valued resources. In this context, secondary trauma symptoms may deplete therapists’ psychological resources (Singh et al., 2020), reducing their ability to utilize meaning in work as a protective factor. Under certain conditions, the positive effects of finding meaning in work may be diminished by the excessive emotional demands placed on therapists (Soren and Ryff, 2023). Moreover, individuals who experience their work as meaningful but are unable to fully utilize their skills and abilities may face a heightened risk of poorer well-being (Allan et al., 2020).

In contrast, sense of hope was associated with lower stress levels, irrespective of secondary trauma symptoms. Hope has been recognized as a significant predictor of psychological adjustment in challenging life situations (Deniz et al., 2023) and serves as an independent protective factor for resilience (Pharris et al., 2022). Current empirical evidence also highlights hope’s strong association with positive psychological functioning and a reduced prevalence of mental health symptoms, including PTSD (Long, 2022). By fostering a sense of agency and goal-directed thinking, hope may help therapists manage stress, even in the presence of secondary trauma symptoms (Passmore et al., 2019).

In addition, in line with COR theory, the current study revealed that therapists who experienced a loss of a loved one during the war exhibited higher levels of stress than did those who did not experience this kind of loss. An association has been found in the literature between therapists who have a history of trauma and their poorer mental health as well as negative changes in their self-perception (Leung et al., 2023). The findings of the present study, similar to findings in the existing literature, highlight the need for further research into the lasting effects of personal loss and trauma among therapists in the context of a collective trauma.

We did not find a significant association between the following factors – therapists’ demographics (age, geographic residence, religion), professional characteristics (experience in trauma therapy, caseload, tenure at the Resilience Center), and other war-related characteristics (being evacuated from one’s home)—and reported stress levels in this study. Although this finding suggests that psychological resources and the loss of a loved one may be particularly salient under extreme circumstances (Hobfoll and Hou, 2025), future research in diverse settings is needed to confirm these results and explore potential contextual influences on therapists’ stress levels.

### Limitations and future directions

This study had several limitations. First, the cross-sectional design restricts the ability to establish causal relations between finding meaning in work, sense of hope, secondary trauma symptoms, and stress, particularly given the ongoing trauma and war. Second, the study relied on self-reported data, which may have been influenced by social desirability biases and retrospective inaccuracies. Future research could benefit from incorporating multi-informant reports and in-depth interviews to enhance validity. Third, the sample size was relatively small, which may have limited the ability to detect significant results. However, the sample did consist of a unique population—therapists working in a high-trauma environment—and the effect sizes were large, offering valuable insights that should be explored further in larger, more diverse samples.

Given that the sample was drawn from a single resilience center in close proximity to Gaza, with a predominantly female therapist population (75%), readers should be mindful when generalizing these findings to other geographical regions or to male therapists. An important limitation of this study was the relatively low response rate, which may limit the generalizability of the findings. Although all eligible therapists at the Center were invited to participate, it is possible that those who chose to respond differ systematically from those who did not, potentially introducing a self-selection bias.

The study’s measurement tools had limitations. Although the single-item stress measure has been validated (Elo et al., 2003; Littman et al., 2006), it provides only a coarse assessment (Benuto et al., 2021), potentially resulting in reduced precision. This choice minimized participant burden, but the findings should be interpreted cautiously. Additionally, there was a significant correlation between the stress and secondary trauma symptom measure, raising concerns about conceptual overlap. Although not statistically redundant (e.g., *r* < 0.85; Bagozzi et al., 1991), this overlap may reflect limitations of the single-item measure. Future studies should consider multi-item stress assessments or alternative outcomes, such as behavioral or interpersonal functioning, to better distinguish the constructs.

Finding meaning in work was assessed through the “motivation-for-the-common-good” subscale. However, other dimensions of finding meaning in work may have a different impact on therapists. Moreover, another limitation of this study was the relatively low Cronbach’s alpha (*α* = 0.65) for the “meaning in work” subscale. Although expected, given the subscale’s limited number of items and minor adaptations for context, the results related to “finding meaning in work” should be interpreted with caution due to the measure’s limited internal consistency. Finally, although we examined key resilience and risk factors, other potential moderating or mediating variables, such as prior trauma exposure, coping strategies, organizational support, and other personal resources, were not included and warrant further investigation.

Future research should confirm the findings and examine the mechanisms through which secondary trauma symptoms may weaken the protective effect of finding meaning in work and explore whether specific coping strategies can mitigate this impact. Longitudinal studies could clarify the temporal relations among these variables and assess whether prolonged exposure to a collective trauma interacts with how finding meaning in work and sense of hope function as resilience factors. Additionally, tracking the development of secondary trauma and stress among therapists in highly traumatic areas would provide deeper insights into the long-term effects of working in such environments. Comparing these therapists with those exposed to a collective trauma but not working directly with trauma survivors—or with those practicing in lower-risk settings—could help delineate the unique roles of hope, meaning, and trauma exposure. Finally, future studies should explore broader expressions of distress among therapists, as well as potential pathways for growth and resilience.

Although resilience is shaped by various factors such as social support, self-efficacy, and emotion regulation (Lev et al., 2022), our study focused specifically on “finding meaning in work” and “sense of hope” due to their strong association with personal growth and sustained motivation. These factors have been shown to be especially important for mental health professionals in trauma-related fields (Passmore et al., 2019; Taubman Ben Ari et al., 2024), making them central to our investigation.

The findings of the current study, although exploratory, have important implications for interventions aimed at supporting trauma therapists. Therapists that experience personal loss (i.e., the loss of a loved one) emerged as a significant factor in predicting their levels of stress during a collective trauma. This finding underscores the need to direct focused attention toward therapists who are personally affected by such events. Moreover, secondary trauma symptoms were found to be highly related to stress. These findings underscore the critical importance of early identification of therapists who may be at elevated risk—particularly those coping with personal loss or exhibiting symptoms of secondary trauma. Utilizing tools such as targeted screening, self-report measures, and attentive supervisory observation can help flag individuals who are at risk before the escalation of their stress levels. Early recognition of those at risk is an essential precursor to effective support or intervention, allowing for timely, tailored responses that safeguard therapists’ well-being and professional functioning.

Recently, meaning-centered psychotherapy (MCP)—a brief, evidence-based intervention—has garnered growing interest for its potential to enhance the well-being of healthcare professionals (Rosa et al., 2025). That said, although fostering meaning in work is beneficial, it may not be sufficient to mitigate stress for those experiencing high secondary trauma symptoms. Instead, targeted strategies to reduce secondary trauma, such as supervision, peer support, and trauma-informed self-care practices, should be prioritized (Elwood et al., 2011; Kim et al., 2022). Additionally, promoting hope through resilience-building interventions may provide a stress-buffering effect (Chmitorz et al., 2018). Brief hope interventions (BHIs) have shown promise not only in enhancing hope and reducing distress, but also in supporting overall psychological resilience and well-being across various high-stress professional contexts (Chan et al., 2019; Kwon et al., 2015).

## Data Availability

The datasets presented in this study can be found in online repositories. This data can be found here: https://osf.io/q3vuh/?view_only=cbc9860ccddc4055aaa4f95f6165cc6b.
