# Correction: Beyond meaning: hope and secondary trauma in Israeli therapists after the October 7th massacre

**DOI:** 10.3389/fpsyg.2025.1649364

**Published:** 2025-06-25

**Authors:** Frontiers Production Office

**Affiliations:** Frontiers Media SA, Lausanne, Switzerland

**Keywords:** hope, meaning, secondary trauma, therapists, war

Due to an error during the finalisation of the article, the authors' affiliations were presented incorrectly in the published article.

The corrected affiliations are shown below.

“Sivan George-Levi^1*^, Lir Faverman^1^, Yael Galin-Lonchich^1^, Anat Ben-Gal Dahan^2, 3^ and Rivi Frei-Landau^1^

^1^Department of Psychology, Achva Academic College, Arugot, Israel

^2^Israeli Center for Addiction and Mental Health (ICAMH), Department of Psychology, The Hebrew University of Jerusalem, Jerusalem, Israel

^3^Sderot Resilience Center, Sderot, Israel”

The original version of this article has been updated.

